# Feasibility study of the Care-at-Home Pharmacotherapy Service (CAHPS): pharmacy technician-led home visits for people receiving polypharmacy who are housebound living in socioeconomically deprived areas

**DOI:** 10.1007/s11096-026-02211-2

**Published:** 2026-09-12

**Authors:** Tania Ramos, Louise Cochrane, Amit Verma, Ling Zhang, Andrew Beattie, Chris F. Johnson, Richard Lowrie

**Affiliations:** 1 Pharmacy Services, Glasgow City Health and Social Care Partnership (North East Locality), NHS Greater Glasgow and Clyde, Glasgow, G69 6GA UK; 2 Townhead Medical Practice, Townhead Health Centre, 16 Alexandra Parade, Glasgow, G31 2ES UK; 3 Pharmacy Services, NHS Greater Glasgow and Clyde, Clarkston Ct, 56 Busby Rd, Glasgow, G76 7AT UK

**Keywords:** General practice, Long-term conditions, Primary healthcare, Pharmacist, Pharmacy technician, Polypharmacy

## Abstract

**Introduction:**

People who are housebound frequently miss general practice-based long-term condition (LTC) reviews. Missingness from LTC reviews are associated with poorer health outcomes and higher costs. General practice pharmacy collaboration within primary care teams may help address the housebound missingness care gap.

**Aim:**

To evaluate the feasibility of implementing the Care-at-Home Pharmacotherapy Service, a general practice pharmacy technician-clinical pharmacist domiciliary review model for housebound patients living in areas of socioeconomic deprivation.

**Method:**

Pre-post intervention study, in two primary care general practices serving a socioeconomically deprived locality. Inclusion criteria: adults ≥ 60 years, coded as housebound with ≥ 2 LTCs and ≥ 5 regularly prescribed medications. Exclusions: adults lacking capacity or receiving palliative care. Baseline data: demographics, Scottish Index of Multiple deprivation (SIMD, a national measure of area-level deprivation), number of regular medications, falls frequency, health-related quality-of-life (EQ5D5L) score, and unscheduled care. Pharmacy technician domiciliary visits involved medication review, adherence support, education and physical assessment. A pharmacist provided clinical supervision and clinical review. Pre-post review outcomes were assessed at 3 months.

**Results:**

Twenty-five patients were included (median age 81, range 60–89; 56% female), with 56% living in the most deprived SIMD quintile. In total, twenty-three patients had missed 91 LTC reviews since becoming housebound and were prescribed a median of 14 regular medications (range 9–27). Across 83 encounters with a pharmacy technician, 21 medications were stopped, 19 initiated, and 15 adjusted. Fifteen clinical referrals were generated. Compared with the 3 months before pre-intervention, the total number of self-reported falls during the 3-month follow-up reduced from 8 (range 0–2) to zero (range 0–0). Mean EQ-5D-5L visual analogue score increased from 57 to 62%, and unscheduled care contacts reduced from a total of 56 (range 0–7) to 21 (0–4).

**Conclusion:**

Pharmacy technician-led domiciliary review with support from a clinical pharmacist is feasible and leads to identification of actionable unmet clinical and medication- needs in older adults who are housebound.

## Impact statements

- New models of care are needed to address health inequalities faced by older adults living in areas of deprivation who miss general practice-based LTC reviews due to being housebound.
- Domiciliary visits by pharmacy technicians delivering LTC reviews offers a potentially sustainable new model of care and is feasible.
- Further testing is required in the context of a pilot randomised controlled trial with embedded qualitative and economic evaluations

## Introduction

Life expectancy is increasing globally. In 2019, over one billion people were aged 60+, a figure projected to double by 2050 [1]. In the UK, medical innovation and improvements in public health have driven an increase in life expectancy from 77.9 years in 2000 to 80.1 years in 2021 [2]. Despite this, life expectancy in Scotland, particularly in deprived urban areas of Glasgow, remains among the lowest in the UK [3, 4]. These inequalities are associated with an earlier onset of multimorbidity, polypharmacy, and frailty, occurring 10–15 years sooner than in more affluent populations. They are further compounded by healthcare ‘missingness’ and housebound status [5–7]. In contrast, people living in affluent areas live > 25 years longer [8].

Lindsay et al*.* (2024) describes the concept of ‘missingness’, as a systematic absence of patients from routine care due to structural, socioeconomic, logistical, or psychosocial barriers rather than deliberate disengagement [9–11]. However, missingness may arise from a combination of factors including low health literacy, previous stigmatising encounters, fear of diagnosis, discrimination, mental health barriers, and practical limitations such as transport, finances, and time [9].

There is no single global prevalence estimate for housebound patients because classification varies internationally and remains subjective [12]. Evidence from the United States, Israel, and Spain have similarly identified housebound older adults as a growing population with high levels of multimorbidity, functional impairment and unmet healthcare need [13–16].

Demand for domiciliary care in the UK is growing. In 2024, Age UK estimated that around two million people (18% of the total adult population aged 65 or over) in England had unmet social care needs, highlighting the need for anticipatory community-based and integrated care models [17]. In Scotland, the number of people receiving Care at Home services (*i.e.* personal care, household tasks, mobility aids, telehealth monitoring and daily living support) increased by 5% to 97,585 people in 2023–24, equating to 8.9% of the adult population in Scotland aged 65 or over [18].

These challenges represent another example of the inverse care law, whereby populations with the greatest healthcare need often have the poorest access to care [19]. Likewise, patients living in deprived areas with multiple long-term conditions (LTCs) often experience shorter and less comprehensive general practice consultations [19, 20]. Current healthcare delivery relies on office-based attendance, disproportionately excluding housebound individuals, potentially further exacerbating inequities of care and intensifying morbidity and mortality disparities associated with the ‘Glasgow effect’ [21, 22]. New models for domiciliary care-at-home outreach may therefore represent an important approach to addressing gaps in current services in the context of ageing populations, where increasing clinical complexity means that demand continues to outstrip service capacity [23].

General practice pharmacy teams have demonstrated effectiveness in delivering domiciliary and outreach care across a range of contexts, including post-hospital discharge [24], chronic obstructive pulmonary disease and multimorbidity reviews [25], care for people experiencing homelessness [26], and LTC management [27–29]. When embedded within general practice, pharmacy teams are well positioned to address gaps in care through structured at-home medication optimisation, guideline-aligned LTC review, and mitigation of medication-related risks (*e.g.* falls), as well as freeing GP capacity [30]. To that end, the Care-at-Home Pharmacotherapy Service (CAHPS) was developed. CAHPS is a pharmacy service-led model embedded within general practice that integrates clinical pharmacists and pharmacy technicians within multidisciplinary team (MDT) structures to provide personalised, at-home LTC and polypharmacy reviews.

Previous domiciliary pharmacy studies have primarily evaluated pharmacist-led interventions [24, 25]. However, increasing multimorbidity, workforce pressures, and growing levels of unmet need among housebound populations have created interest in task-sharing approaches within primary care MDTs. CAHPS was developed to explore whether a pharmacy technician-clinical pharmacist collaborative model could support anticipatory medication-related and LTC care delivery within routine general practice.

### Aim

To evaluate the feasibility of implementing the Care-at-Home Pharmacotherapy Service (CAHPS), a general practice pharmacy technician-clinical pharmacist domiciliary review model for housebound patients living in areas of socioeconomic deprivation.

## Method

### Study design and setting

A pre-post feasibility study, reported in accordance with TIDIeR (Template for Intervention Description and Replication) guidance [31].

NHS Scotland comprises 14 regional Health Boards, each divided into Health and Social Care Partnerships (HSCPs). HSCPs are joint NHS-local authority bodies responsible for planning and delivering integrated community health and social care services. NHS Greater Glasgow and Clyde (NHSGGC) contains six HSCPs. Two general practices serving a socioeconomically deprived population of 35,148 within Glasgow’s North East HSCP, as defined by the Scottish Index of Multiple Deprivation (SIMD), participated from March 2024 to March 2025.

### Patient identification and inclusion

Housebound patients were identified through general practice electronic records. Inclusion criteria: age ≥ 60 years, coded as housebound, living with ≥ 2 LTCs recorded on general practice disease registers; and prescribed ≥ 5 regular medications, defined as medication intended for continuous management of LTCs (e.g. diabetes), excluding short-term treatments, e.g. acute illness. Exclusion criteria: patients receiving palliative care, lacking capacity, previously declining review, or if records indicated a risk of physical harm to staff.

### Intervention

The intervention was delivered by an experienced general practice pharmacy technician in collaboration with a general practice clinical pharmacist who provided clinical supervision and mentoring. The pharmacy technician and pharmacist were embedded within the practice multidisciplinary team, where their routine roles included medication reconciliation, polypharmacy review and chronic disease management support. The pharmacy technician and pharmacist had 12 and 16 years of professional experience, respectively. Pharmacy technicians are a regulated UK workforce by the General Pharmaceutical Council, requiring completion of two years of supervised competency-based vocational training [32, 33]. With over 25,000 technicians currently registered in the UK [34], their traditional roles have focused on dispensing, medication reconciliation, and delegated clinical support; however, in response to service pressures, their contribution to direct patient care is expanding, particularly within primary care settings [32, 33]. The pharmacy technician held a diploma in clinical pharmacy services and therapeutics, and completed phlebotomy and physical examination training prior to CAHPS inception. The pharmacist held additional postgraduate advanced clinical pharmacy qualifications, and was an active independent prescriber. Clinical oversight was provided by practice GPs, and a set of clinical parameter thresholds were agreed with the MDT to guide the decision-making and actions, including escalation to the clinical pharmacist or GP (Table 1).

**Table 1:** Multidisciplinary team-agreed clinical parameter thresholds

	Target ranges	Trigger for on-call GP or clinical pharmacist referral	Reference
Blood pressure	General target: < 140/80 mmHg T2DM: < 135/85 mmHg T2DM with CKD: < 130/80 mmHg Higher targets accepted in frailty	> 160/100 mmHg < 100/60 mmHg Or showing symptoms of postural hypotension (*e.g.* dizziness and light-headedness)	[53–55]
Pulse	Normal range: 60–100 beats per minute	Outside of normal range or irregular	[56]
Oxygen saturation	Normal range: 94–98% room air	92% or less room air	[57, 58]
Respiratory rate	Normal range: 12–20 breaths per minute	> 20 breaths per minute < 12 breaths per minute	[59]
6-CIT	Normal: ≤ 7 (out of 28)	≥ 8 (out of 28)	[38]

*6-CIT* six-item cognitive impairment, *CKD* chronic kidney disease, *T2DM* type 2 diabetes mellitus

CAHPS was delivered in addition to routine general practice clinical duties. The pharmacy technician conducted the home visits during one 3.5-h session (0.1 whole-time equivalent) per week. Clinical review and implementation was delivered by a clinical pharmacist via a telephone call, scheduled on an existing in-practice clinic.

**Fig. 1:**
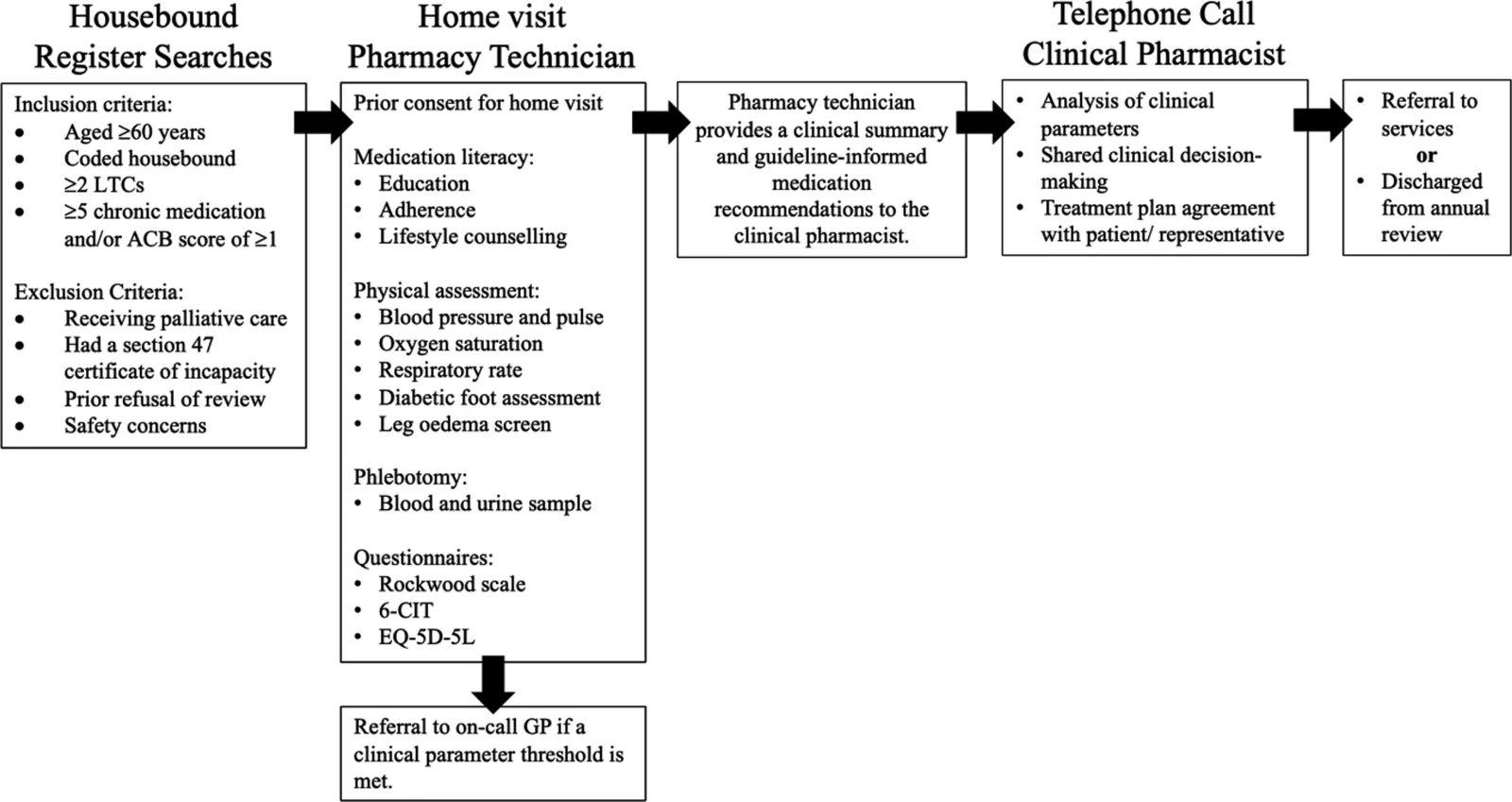
Care-at-home pharmacotherapy service workflow. 6-CIT: six-item cognitive impairment; ACB: anticholinergic cognitive burden; GP: general practitioner. LTCs: long term conditions

Housebound patients were contacted by the pharmacy technician to arrange and agree a home visit review (Fig. 1). During each visit the pharmacy technician followed the polypharmacy 7-steps process to deliver a structured medication review, including: lifestyle counselling, assessments of medication adherence and patient understanding of their treatment indications [35]. Medication adherence was assessed subjectively by identifying medications that were being taken or not taken. Where adherence was poor, patients were educated on the purpose and importance of their prescribed medications [35]. Anticholinergic burden (ACB) score was determined to quantify exposure to anticholinergic medications associated with adverse outcomes in older adults, including falls and cognitive impairment [36]. Self-reported falls and unscheduled-care contacts (i.e. GP out-of-hours contacts, accident and emergency attendance, and hospital admissions) during the preceding 3 months were also recorded.

Primary care-validated assessment tools were used: 6-CIT for cognitive impairment screening, and EQ-5D-5L for health-related quality of life visual analogue scale (VAS) and a 5-dimension descriptive system comprising of mobility, self-care, usual activities, pain/discomfort, and anxiety/depression dimensions. [38, 39]. These measures are not routinely collected during general-practice LTC reviews; however, they were included to help identify medication-related risks for older adults that may present as impaired cognition, reduced mobility, and difficulties with activities of daily living [40, 41].

Physical health assessment included: blood pressure, pulse, oxygen saturation, respiratory rate, lower-limb oedema screening, blood analysis (urea and electrolytes, liver function test, thyroid function test, lipid profile, glycated haemoglobin, HbA1c, and full blood count), urine albumin creatinine ratio (ACR) and frailty assessment using the Rockwood Clinical Frailty Scale [37]. Diabetic patients received an additional foot assessment including monofilament test, peripheral pulse assessment, and hygiene review in line with Diabetes UK [42]. When predefined thresholds were breached (Table 1), the on-call GP was contacted for same-day assessment.

Following the home visit, all cases were discussed with the pharmacist prior to the scheduled telephone consultation (Fig. 1). The pharmacist contacted all patients 7–10 days after the pharmacy technicians’ home visit to review findings, interpret results (including urine and blood tests), and discuss the patient’s clinical and social context (e.g. dexterity, social needs, family or carers involvement). The pharmacist's telephone call with the patient followed the principles of patient-centred shared decision-making and valued-based care [43, 44]. Additional pharmacist follow-up was undertaken whenever medication changes required reassessment, supported by technician domiciliary visits where appropriate, for example blood pressure monitoring following antihypertensive therapy changes (typically at two weeks), HbA1c measurement 3 months after hypoglycaemic therapy adjustments, or whenever further treatment optimisation was indicated. All medication-related decisions were informed by frailty status in accordance with national frailty prescribing guidance [45–47]. Secondary-care referrals were initiated by the pharmacist and authorised by a GP, where appropriate.

Following discussion with the patient, agreed medication changes, referrals and monitoring plans were documented in the patient’s clinical record and implemented in accordance with routine general practice procedures.

At 3 months, medication adherence, self-reported falls, health-related quality of life using the EQ-5D-5L (dimensions and VAS), and unscheduled-care contacts were reassessed during the final pharmacy technician telephone follow-up using the same approach as at baseline. ACB score was recalculated from the updated medication record.

Feasibility was explored through identification of eligible patients, delivery of domiciliary reviews within routine practice, follow-up completion, MDT integration, and collection of service and patient-reported outcome measures.

### Data collection and analysis

Patient-level data was collected in a specifically designed and piloted Excel spreadsheet and anonymised prior to analysis. Baseline collection included: age, sex, postcode-derived Scottish Index of Multiple Deprivation (SIMD) rating [11], number LTCs, number of regular medication and ACB score [36]. Unscheduled-care contacts conducted 3 months prior to review, and missed annual LTC reviews (e.g. diabetes, cardiovascular disease, asthma, chronic obstructive pulmonary disease), were identified from practice electronic records and disease register recall systems, respectively. At the pharmacy technician’s home visit, before any medication changes were made, medication adherence, self-reported falls during the preceding 3 months, Rockwood scale [37], 6-CIT [48], and EQ-5D-5L [37], were collected.

At 3 months post-intervention, medication adherence, self-reported falls, secondary-care referrals, and EQ-5D-5L were reassessed during the final pharmacy technician telephone consultation using the same approach as baseline collection. Practice electronic health records provided the number and type of CAHPS encounters (home visits and telephone calls), number of medication changes, medication adherence status, resulting number of regular medication, ACB score, and number of unscheduled-care contacts.

Medication changes were categorised as: (1) ‘deprescribed’ meaning discontinuation of a regular medication; (2) ‘initiated’ meaning commencement of a new regular medication; and (3) ‘dose-adjusted’ meaning any change in strength or frequency of an existing regular medication. Switches within a therapeutic class (*e.g.* angiotensin converting enzyme inhibitor to angiotensin 2 receptor blocker) were recorded as deprescribed and initiated, respectively. Short-term treatments were included in these counts when medication changes occurred. Estimated changes in prescribing-cost were estimated using NHS Scotland Drug Tariff July 2025, based on a standard 28-day prescribing cycle and annualised to reflect long-term medication use. Short-term treatments were included in the medication-change counts and costs were calculated according to the exact intended duration of therapy.

Clinical observations, blood and urine investigations were collected to support clinical decision-making during the intervention and were not included as study outcome measures.

Pre- and post-intervention data were assessed and summarised as descriptive statistics (means, medians, standard deviations, and confidence intervals, where appropriate), in line with previous studies [52, 53]. Minitab, LLC® was used to summarise data. No formal statistical testing was undertaken, consistent with guidance for feasibility studies [49].

### Patient and public involvement

Patients and members of the public were not involved in the design, delivery, or dissemination of this service development and evaluation.

### Ethics approval

The NHS West of Scotland Research Ethics Service determined that ethical approval was not necessary due to the service evaluation nature of this study. NHSGGC Caldicott approval was sought and granted for the analysis of anonymised patient-level data.

## Results

Ninety-six patients were identified as being coded as housebound; 58 did not meet inclusion criteria (e.g. palliative care, lacking capacity, age < 60 years, or fewer than two LTCs), while 38 were eligible. Of these, 25 eligible patients were reviewed and the remaining 13 eligible patients were not, due to capacity constraints. No eligible patients declined participation.

The final sample (n = 25) had a median age of 81 years (range 60–89); 56% were female and 56% lived in the most socioeconomically deprived quintile in Scotland (Table 2). Patients had a median of 7 LTCs (range 4-11, Table 3) and were prescribed a median of 14 regular medications (range 9–27) with an ACB score 2 (range 0–8). In the 3 months before intervention, patients had 56 unscheduled-care contacts (range 0–7). Baseline data indicated that 23 patients had missed at least one annual LTC review since becoming housebound, two had become housebound more recently and were not yet overdue, accounting for 91 missed reviews in total (range 0–12). A minority of patients (n = 2) were non-adherent with their medication. Six patients (24%) reported falls in the previous 3 months, accounting for eight falls in total (range 0–3). Median Rockwood Frailty Scale was 6 (range 4–7) with EQ-5D-5L VAS score 57% (range 1–96) (Table 4).

**Table 2:** Baseline patient characteristics

Characteristics	Patients who are housebound (n = 25)
Age, median [range]	81 [60–89]
Female, n (%)	14 (56)
SIMD, n (%)	
SIMD 1 (most deprived)	14 (56)
SIMD 2	6 (24)
SIMD 3	5 (20)
SIMD 4–5 (least deprived)	0
No. Comorbidities, median [range]	7 [4–11]
No. regular medication, median [range]	14 [9–27]
ACB score, median [range]	2 [0–8]
Total of unscheduled care^a^ contacts 3 months prior, n [range]	56 [0–7]
Missed annual reviews:	
Number of patients, n (%)	23 (92)
Number of annual reviews missed [range]	91 [0–12]
Medication adherence, n (%)	
Non-adherent	2 (8)
Adherent	23 (92)
Self-reported falls in the previous 3 months	
Number of falls, n [range]	8 [0–3]
Number of patients that fell, n (%)	6 (24)
Rockwood Frailty Score, median [range]	6 [4–7]
EQ-5D-5L VAS baseline, % [range]	57 [1–96]

^a^Defined as any unplanned healthcare contact including GP out-of-hours contacts, accident and emergency attendance, and hospital admission. SIMD: Scottish index of multiple deprivation. ACB: anticholinergic cognitive burden score

**Table 3:** List of long-term conditions

Long-term conditions	n (%)
Type 2 Diabetes Mellitus and hypothyroidism and osteoporosis	18 (72)
Hypertension	15 (60)
Chronic Obstructive Pulmonary Disease	13 (52)
Atrial Fibrillation	12 (48)
Osteoarthritis	12 (48)
Mental Health (*e.g.* anxiety, depression, personality disorder or panic disorder)	10 (40)
Heart Failure	9 (36)
Ischaemic Heart Disease	8 (32)
Chronic Kidney Disease	8 (32)
Previous Myocardial Infarction	7 (28)
Previous Stroke or Peripheral Vascular Disease	6 (24)
Asthma	6 (24)
Gout	5 (20)
Others (Polymyalgia rheumatica, lupus, cognitive impairment and epilepsy)	5 (20)

**Fig. 2:**
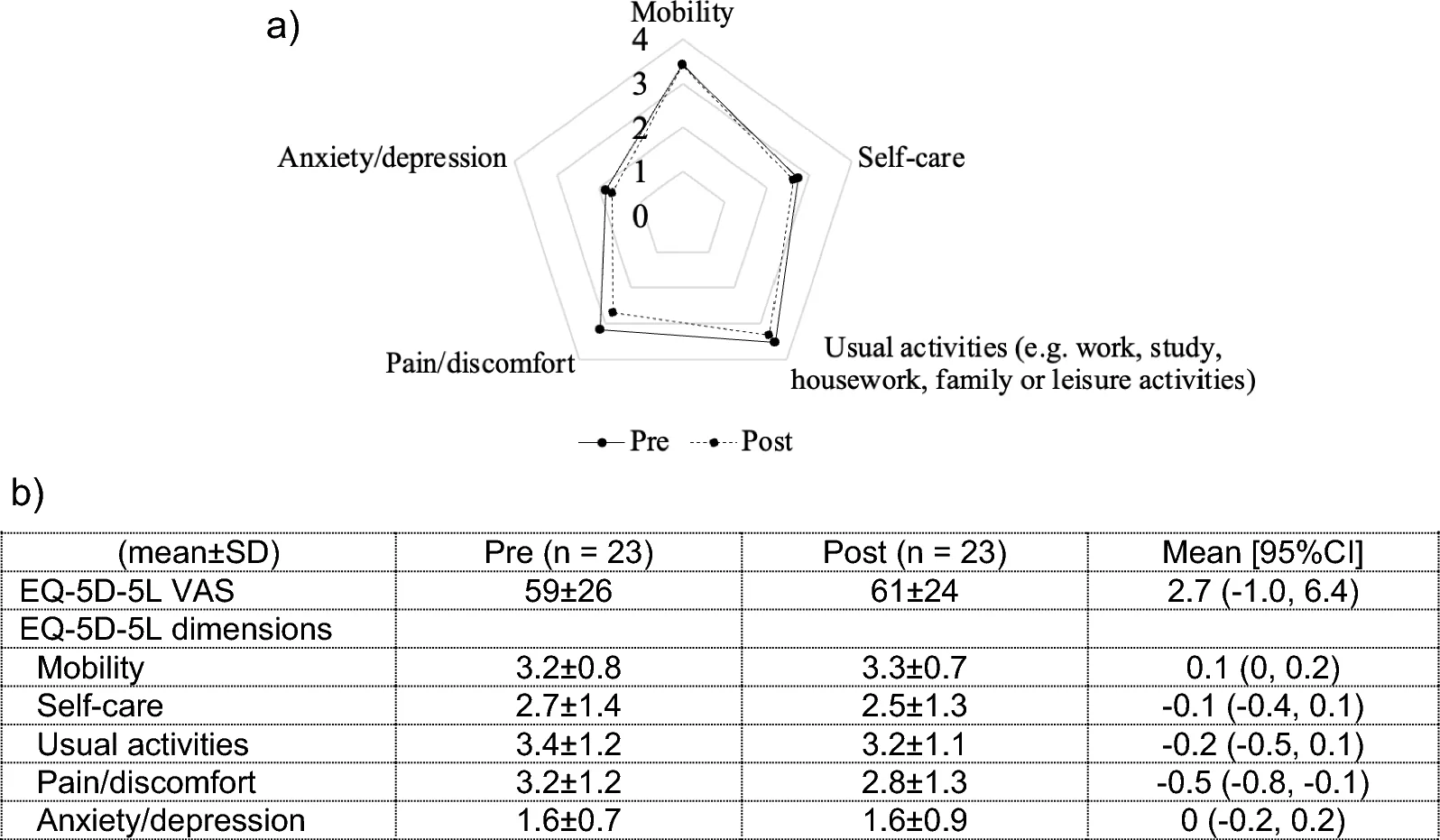
Pre-post EQ-5D-5L dimension-level analysis [41]. **a** Distribution of EQ-5D-5L Visual Analogue Scale dimension scores at baseline and three-month follow-up. **b** Mean ± standard deviation (± SD) pre- and post-intervention

Table 4 and Fig. 2 depict the 3-month pre- and post- results of CAHPS intervention. The pharmacy technician and clinical pharmacist reviews involved 83 patient encounters: 31 home visits and 52 telephone consultations. Structured pharmacotherapy reviews resulted in 21 medications being discontinued, 19 initiated, and 15 dose-adjusted. No adverse events related to the intervention were identified or reported during study period. No change in overall medication burden was observed, however there was an estimated £5083 per annum reduction in prescribing costs due to deprescribing and medication rationalisation, and a £1921 increase due to medication initiation or optimisation, resulting in a net annual reduction of £3162 per annum equating to £126 per patient per annum. Common medication changes included deprescribing or medication rationalisation for: diabetes, gastrointestinal conditions, psychotropics, and related anticholinergic burden, alongside initiation of guideline-directed cardiovascular therapies, anticoagulation, gastroprotection, and vitamin supplementation. There was little impact on overall ACB scores. Median ACB score remained 2 before and after the intervention. Scores ranged from 0–8 at baseline and 0–7 at follow-up. Eighteen patients had unchanged scores, 4 decreased and 3 increased. Unscheduled-care contacts reduced from 56 (range 0–7 per patient) to 21 (range 0–4 per patient) 3 months pre- to post-intervention. Across individual patients, unscheduled-care use decreased in 19 patients, remained unchanged in 5, and increased in 1.

**Table 4:** Pre-post intervention results

Measurement	Pre (25 patients)	Post (25 patients)
Medication		
Discontinued, n	–	21
Initiated, n	–	19
Dose-adjusted, n	–	15
No. regular medication, median [range]	14 [9–27]	14 [8–25]
Medication adherence, n (%)		
Non-adherent	2 (8)	0
Adherent	23 (92)	25 (100)
ACB score, median [range]	2 [0–8]	2 [0–7]
Self-reported falls		
Number of falls, n [range]	8 [0–3]	0
Number of patients that fell, n (%)	6 (24)	0
Total of unscheduled care contacts, n [range]	56 [0–7]	21 [0–4]
Referrals	–	15

*ACB*: anticholinergic cognitive burden score

Medication adherence was subjectively measured and appeared to improve over the follow-up period for the non-adherent patients; achieving full adherence across all 25 patients. Self-reported falls reduced from eight falls, by six patients, to zero, pre- to post-intervention. No new falls were reported by other patients during follow-up period. All 25 patients completed baseline 6-CIT screening. One patient scored ≥ 8 and, supported by clinical concern, was referred to the Memory Assessment Clinic. Overall CAHPS anticipatory care reviews generated 15 referrals for 13 patients: cardiology (n = 3), renal services (n = 1), lipid clinic (n = 1), endocrinology (n = 1), podiatry (n = 3), memory assessment (n = 1), primary care mental health (n = 1), urology (n = 1), social services (n = 1), acute assessment (n = 1), and the on-call GP (n = 1). Twenty-three patients provided pre-post EQ-5D-5L scores (Fig. 2); two declined. Overall, mean self-reported quality of life index score increased from 57% at baseline to 62% at follow-up. At dimension level, pain/discomfort showed the greatest improvement (mean change − 0.5; 95% CI − 0.8 to − 0.1). Usual activities demonstrated a modest improvement (− 0.2; 95% CI − 0.5 to 0.1). Mobility and self-care showed minimal directional change (mean changes + 0.1 and − 0.1, respectively), and anxiety/depression remained unchanged (0; 95% CI − 0.2 to 0.2) (Fig. 2).

## Discussion

### Statement of key findings

This feasibility study demonstrated that a collaborative general practice pharmacy technician-clinical pharmacist model, embedded within the MDT, could be delivered to provide domiciliary LTC reviews and address medication-related needs among housebound older adults.

Patients received timely domiciliary assessments that resulted in medication optimisation, including appropriate deprescribing. There was a signal of fewer unscheduled-care contacts, improved self-reported adherence to prescribed medication, a reduction in self-reported falls, a modest increase in self-reported quality of life, and timely referrals to additional services. While there was no observed change in median ACB score, structured cognitive screening and 6-CIT use, enabled identification of patients with cognitive impairment that may otherwise have gone unrecognised.

A CAHPS model of care may help identify housebound patients whose medication and chronic disease management needs may otherwise go unmet [9] through anticipatory, person-centred care delivered directly in the home environment.

Our findings align with international evidence from Australia, Asia, United States and Europe, demonstrating that domiciliary services can identify substantial medication-related problems among older adults, yet these have shown large variations in clinical benefit [50]. Likewise, UK-based studies have demonstrated mixed findings reporting improvements in medication optimisation, adherence, and falls reduction [25, 27, 28, 52]. A UK study involving four general practices in Leeds found the intervention to be cost-neutral to cost-saving [53]. Evidence from the Home-based Older People’s Medication Review (HOMER) trial, where pharmacist conducted home medication reviews for older people discharged from hospital, did not reduce hospital admissions and was associated with increased readmissions [54]. Subsequent analyses suggested that limited integration with general practice and lack of multidisciplinary governance may have influenced these outcomes, highlighting the importance of context and service design.

### Strengths and weaknesses

The anticipatory approach adopted within CAHPS aligns with the preventative and community-focused priorities set out in the Chief Medical Officer for Scotland 2024–2025 report and NHS England Neighbourhood Health Guidelines 2025/2026 [43, 51]. Implementation barriers included logistical factors such as parking, travel time and laboratory blood-sample collection windows, with morning-only visits required to ensure timely processing. The service was delivered within a single HSCP using a small pragmatic sample constrained by staffing capacity. Exclusion criteria removed 58 patients (60% of those coded housebound), which may have limited reach and equity of access. Under-coding is a recognised challenge in primary care and likely contributed to under-identification of eligible individuals [52]. Measures used did not directly assess clinical disease progression, and observed changes in patient-reported outcomes (e.g. EQ-5D-5L) and unscheduled-care contacts should therefore be interpreted cautiously as potential signals rather than evidence of effect, particularly given the small sample size, uncontrolled pre-post design, and possible influence of external factors or regression to mean. Formal acceptability assessment of patients or staff was not undertaken.

These practical constraints illustrate real-world challenges associated with delivering outreach models and underline the need for supportive infrastructure and flexible care delivery models. The resource intensity of the model may limit scalability without additional workforce and infrastructure support.

### Further research

Following the MRC framework for testing complex interventions, subsequent work should evaluate the effectiveness and cost-avoidance associated with the CAHPS model using controlled study designs with embedded qualitative and health economic evaluation.

## Conclusion

A pharmacy technician-led domiciliary review model within a GP/pharmacist-supervised framework was feasible, meriting further exploration with an implementation trial.

## Data Availability

No datasets were generated or analysed during the current study.
